# Extract of Araçá-Boi and Its Major Phenolic Compound, Trans-Cinnamic Acid, Reduce Viability and Inhibit Migration of Human Metastatic Melanoma Cells

**DOI:** 10.3390/nu16172929

**Published:** 2024-09-01

**Authors:** Felipe Tecchio Borsoi, Gilnei Bruno da Silva, Daiane Manica, Margarete Dulce Bagatini, Glaucia Maria Pastore, Henrique Silvano Arruda

**Affiliations:** 1 Department of Food Science and Nutrition (DEPAN), School of Food Engineering (FEA), University of Campinas (UNICAMP), Monteiro Lobato Street 80, Campinas 13083-862, SP, Brazil; felipe.tecchio@gmail.com (F.T.B.); glaupast@unicamp.br (G.M.P.); 2 Multicentric Postgraduate Program in Biochemistry and Molecular Biology, State University of Santa Catarina (UDESC), Lages 88520-000, SC, Brazil; gilneibrunosilva@gmail.com; 3 Postgraduate Program in Biochemistry, Federal University of Santa Catarina (UFSC), Florianópolis 88040-900, SC, Brazil; daianemanica2011@hotmail.com; 4 Postgraduate Program in Biomedical Sciences, Federal University of Fronteira Sul (UFFS), Chapecó 89815-899, SC, Brazil; margaretebagatini@yahoo.com.br

**Keywords:** *Eugenia stipitata*, skin cancer, Brazilian fruits, polyphenols, anti-cancer effect, phenolic acids, flavonoids, oxidative stress, caspase-3, inflammasome

## Abstract

Cutaneous melanoma is an aggressive type of skin cancer that is recognized for its high metastatic potential and the challenges it presents in its treatment. There has been increasing interest in plant extracts and their potential applications in melanoma. The present study aimed to investigate the content of individual phenolic compounds in araçá-boi extract, evaluate their antioxidant activity, and explore their effects on cell viability, migration properties, oxidative stress levels, and protein expression in the human metastatic melanoma cell line SK-MEL-28. HPLC-DAD analysis identified 11 phenolic compounds in the araçá-boi extract. Trans-cinnamic acid was the main phenolic compound identified; therefore, it was used alone to verify its contribution to antitumor activities. SK-MEL-28 melanoma cells were treated for 24 h with different concentrations of araçá-boi extract and trans-cinnamic acid (200, 400, 600, 800, and 1600 µg/mL). Both the araçá-boi extract and trans-cinnamic acid reduced cell viability, cell migration, and oxidative stress in melanoma cells. Additionally, they modulate proteins involved in apoptosis and inflammation. These findings suggest the therapeutic potential of araçá-boi extract and its phenolic compounds in the context of melanoma, especially in strategies focused on preventing metastasis. Additional studies, such as the analysis of specific signaling pathways, would be valuable in confirming and expanding these observations.

## 1. Introduction

Cutaneous melanoma is an aggressive type of skin cancer that originates from malignant transformations and uncontrolled proliferation of melanocytes, pigment-producing cells of the skin [1]. It poses significant challenges due to its ability to metastasize, leading to poor prognosis if not detected and treated early [2]. The global incidence of melanoma continues to rise steadily, with 331,722 new cases and 58,667 deaths reported in 2022 alone, underscoring its growing public health concerns [3]. The development of melanoma is multifactorial and arises from the interaction between genetic susceptibility and environmental exposure. However, sixty to seventy percent of melanomas are thought to be caused by ultraviolet (UV) radiation from sunlight, which induces DNA mutations in skin cells [4].

Current treatment options for cutaneous melanoma include surgery, radiotherapy, chemotherapy, immunotherapy, and targeted therapy [5]. Cancer immunotherapies and targeted therapies have been gaining increasing attention with the development of immune checkpoint blockade strategies targeting programmed death-1 (PD-1) and cytotoxic T lymphocyte antigen-4 (CTLA-4) co-inhibitory receptors and mitogen-activated protein kinases (MAPKs) molecular targeted therapy directed at oncogenic serine/threonine-protein kinase B-Raf (BRAF) and mitogen-activated extracellular signal-regulated kinase (MEK) signaling pathways [4,5]. Although the emergence of immune and targeted therapies has increased the life expectancy of patients with melanoma, some patients relapse or simply do not respond to these regimens. Therefore, the development of new and improved adjuvant therapies or drugs remains a priority for researchers to improve patient survival and health quality [1].

In recent years, there has been growing interest in plant extracts, particularly those rich in phenolic compounds, for their potential applications in cutaneous melanoma [6,7,8,9,10]. Phenolic compounds are a group of naturally occurring organic substances found in the plant kingdom as secondary metabolites and are characterized by the presence of hydroxyl (–OH) groups attached to an aromatic hydrocarbon ring. This structural characteristic allows antioxidant properties and can interact with cellular signaling cascades, regulating the activity of transcription factors and consequently affecting the expression of genes involved in cellular metabolism and cell survival, mainly related to cell growth, apoptosis, and inhibition of metastasis [10,11,12,13,14].

Araçá-boi (*Eugenia stipitata* Mac Vaugh-Myrtaceae) is a fruit tree that grows naturally in the Amazon region and is cultivated in some countries such as Brazil, Peru, Bolivia, Ecuador, and Colombia. It is a fleshy globe-shaped fruit, approximately 12 cm in diameter and weighing around 30–80 g, characterized by a fine, yellow, pubescent epicarp, white soft mucilaginous pulp, very acidic (pH = 2.28), and an attractive flavor [15]. In the literature, data on the characterization and quantification of phenolic compounds do araçá-boi, and their biological effects are still scarce. Some studies reported the presence of quercetin, myricetin, kaempferol, luteolin, gallic acid, vanillic acid, apigenin, catechin, gallocatechin, and their derivatives [15,16,17,18]. Additionally, few studies have demonstrated the biological activities of araçá-boi extract, including antioxidant, antimutagenic, antigenotoxic, and anti-inflammatory activities [16,18]. Thus, demonstrating the antitumor activity of araçá-boi could be a tool for managing public health issues, as the inclusion of araçá-boi or its derived products (e.g., juices, extracts, and processed products) in the diet could contribute to the intake of potentially anticarcinogenic phytochemicals, particularly phenolic compounds. This approach may not only play a role in prevention but also serve as a promising therapeutic strategy for the treatment of melanoma. In this context, the present study aims to investigate the content of individual phenolic compounds in araçá-boi extract by high-performance liquid chromatography coupled to a photodiode array detector (HPLC-DAD) and explore their effects on cell viability, migration properties, oxidative stress levels, and protein expression in the human metastatic melanoma cell line SK-MEL-28.

## 2. Materials and Methods

### 2.1. Chemicals and Reagents

Folin-Ciocalteu reagent, 6-hydroxy-2,5,7,8-tetramethylchroman-2-carboxylic acid (Trolox), 2,2′-azobis(2-methyl propionamidine)-dihydrochloride (AAPH), 2,2′-azinobis-(3-ethylbenzothiazoline-6-sulfonic acid) diammonium salt (ABTS), fluorescein, 2,4,6-tripyridy-*s*-triazine (TPTZ), acetonitrile and formic acid grade HPLC, and all phenolic compound standards (gallic acid, α-resorcylic acid, protocatechuic acid, procyanidin B1, chlorogenic acid, catechin, procyanidin B2, 4-hydroxybenzoic acid, gentisic acid, epicatechin, caffeic acid, vanillic acid, syringic acid, vitexin-2-rhamnoside, rutin, vitexin, quercetin-3-*O*-galactoside, *p*-coumaric acid, procyanidin A2, sinapic acid, kaempferol-3-*O*-glucoside, ferulic acid, quercetrin, apigenin-7-*O*-glucoside, myricetin, benzoic acid, luteolin, quercetin, trans-cinnamic acid, apigenin, naringenin, kaempferol, and hesperetin) with a purity of ≥96% were purchased from Sigma-Aldrich (St. Louis, MO, USA). All materials used for cell culture were obtained from Gibco™ Thermo Fisher Scientific (Grand Island, NY, USA) and Invitrogen Life Technologies (Carlsbad, CA, USA). antibodies anti-human *Caspase-3* (p17 subunit; catalog no. BS-20363R-A555), and anti-human *NLRP3* (catalog no. MA5-32255) was purchased from Invitrogen Life Technologies (Carlsbad, CA, USA).

### 2.2. Plant Material and Phenolic Extract Obtantion

Mature araçá-boi fruits were collected from the “Kamui Farm” in Ituberá city, Bahia State (Brazil), at 13°44″ S and 39°9″ W. Botanical identification and exsiccate (access number 55.875) were deposited at the Herbarium-UEC of the Agronomic Institute of Campinas, State of São Paulo, Brazil [19]. The edible fractions (pulp and peel) were freeze-dried (Lyophilizer Series LS E, Terroni Scientific Equipment, São Carlos, SP, Brazil) and ground using a knife mill (Marconi, model MA340, Piracicaba, SP, Brazil). The powders obtained were granulometrically standardized using an electromagnetic sieve shaker 24-mesh (Bertel, model AGMAGB, Caieiras, SP, Brazil).

The phenolic extract was obtained according to the method described by de Araújo et al. [15] with slight modifications. A hydroethanolic solution was selected as the extractor solvent because, among the organic solvents suitable for the extraction of phenolic compounds, only ethanol is considered a generally recognized as safe (GRAS) solvent. Furthermore, its combination with water increases the efficiency of phenolic extraction, particularly in the glycosylated fraction. Thus, the use of an ethanol-water mixture (80:20, *v*/*v*), along with a non-conventional extraction technique (ultrasound), was employed in the present study to address environmental and safety concerns [20,21].

Freeze-dried samples of araçá-boi (1 g) were extracted with 15 mL of an ethanol-water mixture (80:20, *v*/*v*) for 10 min using an ultrasonic bath (UNIQUE, model UCS-2850, 25 kHz, 120 W, São Paulo, SP, Brazil) at room temperature. After extraction, the mixture was centrifuged at 4000× *g* for 5 min, and the upper layer was collected. The residues were re-extracted two more times under the same conditions. The upper layers were combined, evaporated under a vacuum, and freeze-dried. The obtained powder extract was stored at −20 °C until further analysis.

### 2.3. Chemical Characterization of Araçá-Boi Extract

#### 2.3.1. Determination of Total Phenolic Content (TPC)

Total phenolic content (TPC) was determined using the method described by Pereira et al. [22]. The extract (25 µL), 50% (*v*/*v*) Folin-Ciocalteu reagent (25 µL), and 5% (*w*/*v*) sodium carbonate (200 µL) were mixed and allowed to react for 20 min in the dark at room temperature. The absorbance was recorded at 760 nm using a microplate reader (SPECTROstar Nano, BMG Labtech, Ortenberg, Germany). Gallic acid was used as a standard, and the results were expressed as mg gallic acid equivalents per gram of dried extract (mg GAE/g dw).

#### 2.3.2. Determination of Condensed Tannin Content (CTC)

The condensed tannin content (CTC) was determined according to the method described by Arruda et al. [23] with slight modifications. The extract (20 µL), 4% (*w*/*v*) vanillin in methanol (180 µL), and concentrated HCl (90 µL) were mixed and allowed to react for 20 min in the dark at room temperature. The absorbance was recorded at 500 nm using a microplate reader (SPECTROstar Nano, BMG Labtech, Ortenberg, Germany). Catechin was used as a standard, and the results were expressed as mg catechin equivalents per gram of dried extract (mg CE/g dw).

#### 2.3.3. Chromatographic Analysis of Phenolic Compounds by HPLC-DAD

The extracts were analyzed following the chromatographic conditions established by Silva et al. [24] using a Dionex UltiMate 3000 (Thermo Fisher Scientific, Waltham, MA, USA). Chromatographic separation was carried out on a reverse-phase AcclaimTM 120 A C18 column (250 × 4.6 mm i.d., 5 μm particle size, Thermo Fisher Scientific, Waltham, MA, USA) and thermostated at 32 °C. The solvents used were 0.1% formic acid in deionized water (Solvent A) and HPLC-grade acetonitrile (Solvent B). The gradient was linear at a flow rate of 0.5 mL/min with 5% solvent B for 5 min, from 5% to 29% solvent B for 22 min, 35% solvent B for 6 min, 35% to 50% solvent B for 12 min, 95% solvent B for 5 min, and 5% solvent B for 10 min. Diode array detection was performed from 200 to 800 nm. The injection volume was 20 μL. Quantification of different phenolic compounds was carried out at different wavelengths depending on the compound (260, 280, 320, or 360 nm). The compounds were identified and quantified based on their retention times and spectral characteristics compared to those of standard compounds. The contents of individual phenolic compounds were expressed as micrograms per gram of dried extract (µg/g dw).

#### 2.3.4. Trolox Equivalent Antioxidant Capacity (TEAC) Assay Using ABTS^•+^ Radical

The TEAC was conducted as described by Arruda et al. [20]. A radical cation ABTS^•+^ solution (7 mmol/L ABTS and 145 mmol/L potassium persulfate) was prepared and incubated in the dark at room temperature overnight. The ABTS^•+^ working solution was diluted with ultrapure water to achieve an absorbance of 0.70 ± 0.02 at 734 nm. The extract (40 µL) was mixed with ABTS^•+^ solution (200 µL) and the absorbance was measured on a microplate reader (SPECTROstar Nano, BMG Labtech, Ortenberg, Germany) after 6 min at 734 nm. A calibration curve with Trolox was used as a reference, and the results were expressed as micromoles of Trolox equivalents per gram of dried extract (µmol TE/g dw).

#### 2.3.5. Ferric-Reducing Antioxidant Power (FRAP) Assay

The FRAP assay was performed based on the method described by Guerra-Ramírez et al. [25] with some modifications. The FRAP solution was prepared by adding 20 mL acetate buffer (0.3 mol/L) at pH 3.6, 2 mL TPTZ solution (10 mmol/L) in 40 mmol/L HCl, and 2 mL ferric chloride solution (20 mmol/L) (10:1:1). The extract (20 µL), FRAP solution (180 µL), and deionized water (60 µL) were mixed and incubated at 37 °C for 30 min before measuring the absorbance at 595 nm using a microplate reader (SPECTROstar Nano, BMG Labtech, Ortenberg, Germany). A calibration curve with Trolox was utilized as a reference, and results were expressed as micromoles of Trolox equivalents per gram of dried extract (µmol TE/g dw).

#### 2.3.6. Oxygen Radical Absorbance Capacity (ORAC) Assay

The ORAC assay was conducted following the protocol reported by Dávalos et al. [26]. The reaction was performed in phosphate buffer (75 mmol/L, pH 7.4) in a 96-well dark microplate. The extract (20 µL), 1 µmol/L fluorescein (120 µL), and 0.4 mol/L AAPH (60 µL) were mixed and incubated at 37 °C. Fluorescence was measured with excitation at 485 nm and emission at 520 nm every minute for 80 min on a microplate reader (NOVOstar, BMG Labtech, Offenburg, Germany). A calibration curve with Trolox was utilized as a reference, and results were expressed as micromoles of Trolox equivalents per gram of dried extract (µmol TE/g dw).

### 2.4. Cellular Assays

#### 2.4.1. Cell Culture and Treatments

The human metastatic melanoma cell line (SK-MEL-28, Cell Bank of Rio de Janeiro, Brazil) was maintained in Dulbecco’s modified Eagle’s medium supplemented with 10% fetal bovine serum and penicillin/streptomycin. Cells were maintained in an incubator at 37 °C and 5% CO_2_. The powdered araçá-boi extract and trans-cinnamic acid were dissolved in 0.4% dimethyl sulfoxide (DMSO) and mixed with culture medium to obtain treatment concentrations of 200, 400, 800, and 1600 µg/mL. The control group (CT) received only a culture medium for treatment.

#### 2.4.2. Cell Viability by Fluorescence Microscopy Assay

The fluorophore acridine orange (AO) was used to stain viable cells due to its ability to permeate the membranes of living cells and interact with DNA, emitting green fluorescence [27]. Melanoma cells were grown until a monolayer reached a density of 1 × 10^5^ cells/well in 96-well plates and treated with araçá-boi extract and trans-cinnamic acid for 24 h. The cells were then washed twice with PBS and stained with acridine orange (1 µg/mL) for 5 s. The reminiscent staining solution was discarded, and the cells were washed twice with PBS. The readings were taken using an inverted fluorescence microscope (Nikon Eclipse TS2-FL, Tokyo, Japan) with a magnification of 200× at the central point of the wells (Ex = 480 nm, Em = 500 nm). The software ImageJ version 1.54j was used to linearly adjust the images for brightness and contrast. The results are expressed as a percentage (%) of cell viability compared to the control.

#### 2.4.3. Measurement of Mitochondrial Transmembrane Potential (ΔΨm)

The cationic dye tetramethylrhodamine ethyl ester (TMRE) was used to measure the mitochondrial transmembrane potential due to its ability to accumulate in active mitochondria, emitting red fluorescence [28]. Melanoma cells were grown until a monolayer reached a density of 1 × 10^5^ cells/well in 96-well plates and treated with the araçá-boi extract and trans-cinnamic acid for 24 h. Then, the cells were washed twice with PBS buffer and incubated for 30 min with a solution of Hank’s balanced salts containing 20 nmol/L TMRE (100 µL). The reminiscent solution was discarded, cells were washed with PBS, and readings were taken under a fluorescence microscope at a magnification of 200× in the center of the wells (Ex = 550 nm, Em = 580 nm). The software ImageJ version 1.54j was used to adjust the images for brightness and contrast linearly. The results are expressed as a percentage (%) of the mitochondrial transmembrane potential compared to the control.

#### 2.4.4. Cell Migration Assay

To verify the influence of the treatments on cell migration, we performed an assay based on the study proposed by Justus et al. [29]. For this, melanoma cells were cultured in a six-well plate at a density of 1 × 10^6^ cells for 24 h. Scratches were made in a confluent monolayer using a sterile tip. Subsequently, the cells were exposed to araçá-boi extract and trans-cinnamic acid for 24 h. The cells were washed with 0.9% NaCl solution to remove nonadherent cell debris. Cells that migrated across the wound area were photographed at 0 and 24 h using optical microscopy at a magnification of 40×. The software ImageJ version 1.54j was used to adjust the images for brightness and contrast linearly. The results are expressed as a percentage (%) of wound healing closure compared to the control.

#### 2.4.5. Detection of Cellular Reactive Oxygen Species (ROS)

To detect cellular reactive oxygen species (ROS) levels in melanoma cells, a cell-permeable fluorescent signal indicator, 2,7-dichlorodihydrofluorescein-diacetate (H_2_DCF-DA), was used, according to Wu et al. [30]. Thus, after araçá-boi extract and trans-cinnamic acid treatments, cells were washed with PBS and incubated with 100 mmol/L H_2_DCF-DA for 30 min at 37 °C. The final fluorescent product was measured using a fluorescence plate reader (Thermo Scientific™ Varioskan™ LUX, Waltham, MA, USA) (Ex = 488 nm, Em = 525 nm). The results are expressed as a percentage (%) of relative fluorescence compared to the control.

#### 2.4.6. Assessment of Caspase-3 and NLRP3 Expression

To assess caspase-3 and NLRP3 expression in melanoma cells, 1 × 10^6^ cells were collected after 24 h of treatment, washed with PBS, and centrifuged at 3000 rpm for 5 min. Then, the cell pellets were diluted in 100 μL of PBS and mixed with 1 μL of each conjugated antibody (CASP3 or NLRP3). After 30 min, the cells were read in a C6 Plus Personal flow cytometer (Accuri™, BD Accuri, San Jose, CA, USA) every 10,000 events. The results are expressed as a percentage (%) of Caspase-3 and NLRP3 expression compared to the control.

### 2.5. Statistical Analysis

The results are expressed as the mean ± standard deviation value of at least three independent experiments. Statistical analyses were performed using the GraphPad Prism software version 9. Significant differences are symbolized using *p*-values of * *p* < 0.05, ** *p* < 0.01, *** *p* < 0.001, and **** *p* < 0.0001, which were acquired using one-way ANOVA, followed by Dunnett’s post-hoc multiple comparison test.

## 3. Results

### 3.1. Chemical Characterization of Araçá-Boi Extract

#### 3.1.1. Total Phenolic Content, Condensed Tannin Content, and Antioxidant Activity

The total phenolic content, condensed tannin content, and antioxidant activity of the araçá-boi extract are shown in Table 1. Araçá-boi extract showed a total phenolic content of 12.16 ± 0.38 mg GAE/g extract dw. Rufino et al. [31] classified food matrices based on their dry weight (dw) as low (<10 mg GAE/g), medium (10–50 mg GAE/g), and high (>50 mg GAE/g). Therefore, the araçá-boi extract can be considered a good source of phenolic compounds since it exhibits a medium content of these phytochemicals. Our study reported higher total phenolic values than those reported by de Araújo et al. [15] (9.06 mg GAE/g extract dw), who also extracted phenolic compounds from araçá-boi using ultrasound-assisted extraction with ethanol-water mixture (80:20, *v*/*v*) solvent system. Variations in total phenolic content across studies can be attributed to factors such as edaphoclimatic conditions of the fruit’s region of origin (e.g., temperature, soil composition, light exposure, and harvest timing), along with the plant’s physiological and genetic traits, as well as differences in sample preparation and storage conditions [32].

The condensed tannin content was estimated in the araçá-boi extract. As shown in Table 1, the amount of extractable condensed tannin content was 5.76 ± 0.07 mg CE/g extract dw. Condensed tannins, also known as proanthocyanidins, are phenolic compounds found in several fruits. They are polymers formed by the condensation of flavan-3-ols, such as catechin and epicatechin [33]. To the best of our knowledge, this is the first study to report the condensed tannin content in araçá-boi extract.

A strong and positive correlation between the total phenolic content/condensed tannin content and the antioxidant activity of different plant matrices has been demonstrated by other researchers [23,34,35]. Antioxidant activity is mainly linked to phenolic compounds present in the plant matrix, acting as reducing agents, hydrogen donors, transition metal chelators, reactive oxygen and/or nitrogen species (ROS/RNS) quenchers, inhibitors of enzymes involved in oxidative stress, and upregulation and/or protection of endogenous defense systems [23]. Thus, considering these mechanisms of action, the antioxidant activity of the araçá-boi extract was determined using TEAC, FRAP, and ORAC assays. As seen in Table 1, araçá-boi extract showed a higher antioxidant activity value in the ORAC assay (583.81 ± 17.67 µmol TE/g extract dw), followed by FRAP assay (150.77 ± 4.37 µmol TE/g extract dw), and TEAC assay (102.51 ± 1.16 µmol TE/g extract dw). Antioxidant methods are based on different mechanisms of action, including hydrogen atom transfer (the ability to quench free radicals by hydrogen donation), single-electron transfer (the ability to transfer one electron to reduce any compound, such as radicals, metals, and carbonyls), or mixed-mode assays. Therefore, to better understand the antioxidant potential of plant extracts, it is necessary to conduct various antioxidant assays to evaluate their different mechanisms of action [23,36,37]. In the present study, we subjected the araçá-boi extract to antioxidant assays involving each of the aforementioned mechanisms of action, namely hydrogen atoms transfer (ORAC), single electron transfer (FRAP), and mixed-mode (ABTS). As observed above, the araçá-boi extract showed the highest antioxidant activity by the ORAC method, followed by FRAP and TEAC, demonstrating that the phenolic compounds of the araçá-boi extract act more efficiently through hydrogen atom transfer mechanisms. Furthermore, the ORAC method measures a compound’s ability to inhibit peroxyl radicals generated by simulating the physiological conditions of the human body more accurately. Peroxyl radicals are a class of highly reactive free radicals formed during oxidation reactions in the human body and play a significant role in oxidative stress, which is associated with various pathological conditions, including cardiovascular diseases, cancer, inflammatory processes, and aging [23]. The effective capacity of the araçá-boi extract as peroxyl radical scavengers found here points to their potential to prevent and/or treat oxidative-related diseases.

#### 3.1.2. Content of Individual Phenolic Compounds by HPLC-DAD Method

To examine the content of individual phenolic compounds in the araçá-boi extract, HPLC-DAD analysis was carried out using thirty-three authentic standards (Table 2). The data were collected according to the retention time and chromatograms were simultaneously recorded at 260, 280, 320, and 360 nm, approaching the optimal wavelength (λmax) for each compound. Eleven phenolic compounds were identified and quantified in the araçá-boi extract, including seven phenolic acids (gentisic acid, 4-hydroxybenzoic acid, ferulic acid, gallic acid, *p*-coumaric acid, syringic acid, and trans-cinnamic acid) and four flavonoids (hesperetin, kaempferol-3-*O*-glucoside, quercetin-3-*O*-galactoside, and quercetin-3-*O*-rhamnoside). A total value of 766.44 ± 4.53 µg/g extract dw of phenolic compounds was quantified in the araçá-boi extract using HPLC-DAD (Table 2). Additionally, about 56% of the total quantified (435.09 ± 2.19 µg/g extract dw) are phenolic acids, and the other 44% were flavonoids (331.35 ± 2.49 µg/g extract dw), demonstrating a diversification of the phenolic classes found. In terms of compounds, trans-cinnamic acid (155.79 ± 0.32 µg/g extract dw) was the major phenolic compound present in the araçá-boi extract, followed by quercetin-3-O-galactoside (151.32 ± 1.86 µg/g extract dw), quercetin-3-*O*-rhamnoside (116.27 ± 0.27 µg/g extract dw), and syringic acid (113.07 ± 0.74 µg/g extract dw), accounting for approximately 70% of the total quantified phenolic compounds. Few studies have been conducted on the identification and quantification of phenolic compounds present in the fractions of the araçá-boi fruit. For example, Cuellar et al. [38] identified and quantified three phenolic acids, such as chlorogenic acid (44.1–515.1 µg/g dw), gallic acid (17.3–64.8 µg/g dw), and caffeic acid (3.0–11.1 µg/g dw) in an acidified methanol solution (80:19:1, *v*/*v*/*v*, methanol-water-HCl) of the epicarp or mesocarp of araçá-boi fruit at different ripening stages. A study conducted by Neri-Numa et al. [18] found three flavonoids, namely myricetin (0.17 µg/g fw), quercetin (0.09 µg/g fw), and kaempferol (0.03 µg/g fw) in an acid-hydrolyzed methanolic extract of araçá-boi pulp.

To the best of our knowledge, the data presented in this study represent the most detailed quantification of the phenolic compounds in the araçá-boi extract. The major phenolic compounds found in araçá-boi extracts have been reported to exert numerous biological effects. For example, trans-cinnamic acid possesses several significant bioactivities, including antioxidant, antimicrobial, anti-inflammatory, anticancer, antidiabetic, neuroprotective, hepatoprotective, antiallergic, and wound healing properties [39,40,41]. Similarly, various studies demonstrate the potential of quercetin and its derivatives in diverse biological activities through several molecular mechanisms, including modulation of oxidative stress pathways, inhibition of inflammatory signaling pathways, and modulation of cancer-related molecular signaling pathways [42,43,44]. Therefore, these findings suggest that araçá-boi extract possesses a broad and varied number of phenolic compounds, allowing for a potential antioxidant and a broad spectrum of bioactivities that can contribute to promoting human health and well-being. Moreover, different phenolic compounds can interact synergistically to enhance their overall activity. Trans-cinnamic acid is the major compound in the araçá-boi extract (corresponding to approximately one-fifth of the total phenolic compounds identified and quantified by HPLC-DAD). Therefore, this compound was chosen for comparison in the subsequent analyses.

### 3.2. Effects of Araçá-Boi Extract and Trans-Cinnamic Acid on Human Metastatic Melanoma Cells

#### 3.2.1. Cell Viability in Melanoma Cells

**Figure 1:**
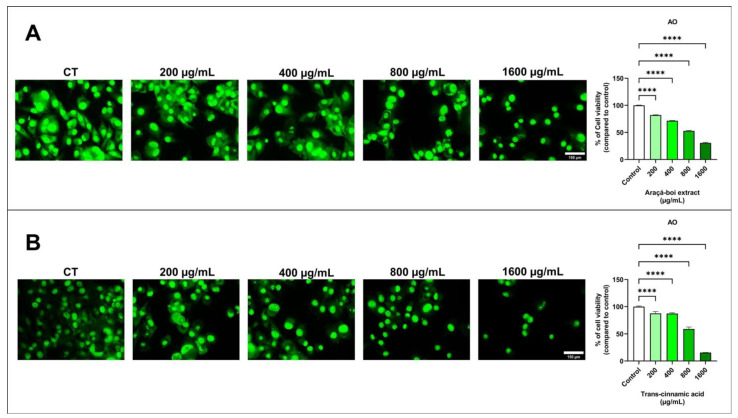
Fluorescence microscopy assay for cell viability using acridine orange (AO) was evaluated in melanoma cells treated with araçá-boi extract (**A**) and trans-cinnamic acid (**B**). CT = control group cells. White scale bar represents 150 μm. Microscope magnification = 200×. All experiments were independently performed three times and in three replicates. Data are presented as the mean ± standard deviation. Statistical analysis: One-way ANOVA followed by post-hoc Dunnett’s multiple comparisons test. Statistical significance was set at *p* < 0.05. **** (*p* < 0.0001).

The viability of SK-MEL-28 cells was assessed by fluorescence microscopy using an acridine orange fluorophore. After 24 h, melanoma cells stained with acridine orange showed a significant decrease in cell viability at all tested concentrations of araçá-boi extract and trans-cinnamic acid compared to the control group cells (*p* < 0.0001) (Figure 1).

Previous studies have highlighted that phenolic compounds present in plant extracts are primarily responsible for their antitumor activities in various types of cancer, including melanoma [10,45,46]. This antitumor activity may be related to the different stages of cancer progression, such as initiation, promotion, progression, invasion, and metastasis [47]. Therefore, to assess antitumor activity, a cell viability assay was performed using fluorescence microscopy, allowing direct observation of membrane integrity on viable cells through the interaction between acridine orange dye and nucleic acids by emitting green fluorescence [48]. As shown in Figure 1A, araçá-boi extract significantly reduced cell viability as the concentration increased. Similarly, this can be observed for trans-cinnamic acid (Figure 1B), indicating that this phenolic acid may be the main contributor to the reduction in melanoma tumor cell viability. Thus, for the first time, the effect of the araçá-boi extract on reducing cell viability in melanoma cells was evaluated, as well as the actual contribution of its major component, trans-cinnamic acid. Several studies have demonstrated that phenolic plant extracts can inhibit cell viability through different mechanisms, such as inducing apoptosis, cell cycle arrest, oxidative stress induction, modulation of signaling pathways, changes in cell membranes, mitochondrial alterations, and DNA damage, among others [47,49]. Therefore, to understand and elucidate the mechanisms by which the araçá-boi extract and trans-cinnamic acid may reduce cell viability, changes in transmembrane potential, oxidative stress induction, and modulation of proteins related to apoptosis and inflammation were performed and are discussed below.

#### 3.2.2. Transmembrane Potential of Mitochondria of Melanoma Cells (ΔΨm)

**Figure 2:**
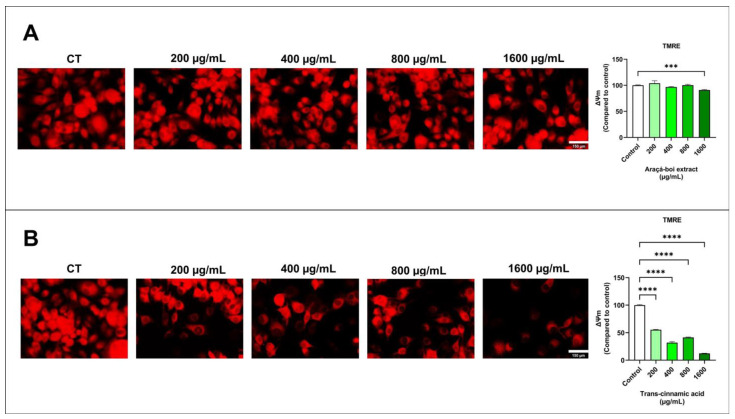
Fluorescence microscopy assay for the transmembrane potential of mitochondria (TMRE) was evaluated in melanoma cells treated with araçá-boi extract (**A**) and trans-cinnamic acid (**B**). CT = control group cells. White scale bar represents 150 μm. Microscope magnification = 200×. All experiments were performed independently three times and in three replicates. Data are presented as mean ± standard deviation. Statistical analysis: one-way ANOVA followed by a post hoc Dunnett’s multiple comparisons test. Values with *p* < 0.05 were considered statistically significant. *** (*p* < 0.001) and **** (*p* < 0.0001).

The transmembrane potential of the mitochondria in SK-MEL-28 cells was assessed by fluorescence microscopy using TMRE (Figure 2). After 24 h, melanoma cells stained with TMRE showed a slight reduction in mitochondrial function with the araçá-boi extract at the highest concentration tested (1600 µg/mL) compared with the control group cells (Figure 2A). In contrast, a noticeable reduction in mitochondrial function was observed with trans-cinnamic acid treatment, as indicated by progressively lower fluorescence intensity at concentrations of 200, 400, 800, and 1600 µg/mL (Figure 2B).

Mitochondria produce adenosine triphosphate (ATP) through oxidative phosphorylation within cells. This metabolic process involves actively transporting positively charged protons across the inner mitochondrial membrane, creating a negative charge known as the mitochondrial transmembrane potential [50]. Dysfunction of the mitochondrial membrane potential in normal cells can induce undesired loss of cell viability and is involved in the onset of several diseases, including chronic respiratory diseases, neurodegenerative diseases, cardiovascular diseases, and cancer [51,52,53,54]. However, this effect is desirable for therapies related to the management and treatment of cancer. Recently, phenolic compounds have been studied for their ability to affect mitochondrial function in cancer cells, specifically by reducing the mitochondrial transmembrane potential [55,56]. Phenolic compounds can induce dysfunction in the mitochondrial transmembrane potential of cancer cells through multiple mechanisms, including induction of oxidative stress, inhibition of the electron transport chain, modulation of apoptotic proteins, and disruption of energy metabolism [57,58,59]. In this study, it was observed that the araçá-boi extract caused a slight reduction in mitochondrial transmembrane potential, while trans-cinnamic acid was visibly more aggressive. These findings lead us to infer that the effect of the araçá-boi extract only at higher concentrations is because the extract is a complex mixture of phenolic compounds (predominantly phenolic acids and glycosylated flavonoids) and non-phenolic compounds (e.g., sugars and organic acids). Consequently, the concentration of trans-cinnamic acid in the extract is diluted among the other phenolic compounds when compared to isolated trans-cinnamic acid, reducing its impact on the mitochondrial transmembrane potential.

#### 3.2.3. Cell Migration of Melanoma Cells

**Figure 3:**
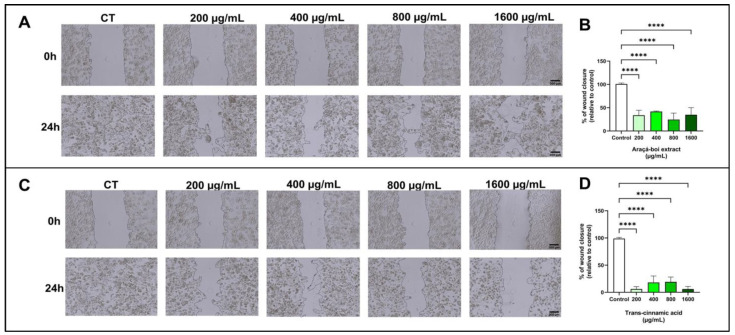
The migration of melanoma cells was evaluated by the wound-healing assay. After cell monolayer formation, a scratch was made, cells were treated with araçá-boi extract (**A**) and trans-cinnamic acid (**C**) for 24 h, and photomicrography was performed. Araçá-boi extract (**B**) and trans-cinnamic acid (**D**) significantly prevented wound closure in the range from 200 μg/mL to 1600 μg/mL. CT = control group cells. Black scale bar represents 300 μm. Microscope magnification = 40×. All experiments were performed independently three times and in three replicates. Data are presented as mean ± standard deviation. Statistical analysis: one-way ANOVA followed by a post hoc Dunnett’s multiple comparisons test. Values with *p* < 0.05 were considered statistically significant. **** (*p* < 0.0001).

Figure 3 shows melanoma cell migration (SK-MEL-28) treated for 24 h with araçá-boi extract and trans-cinnamic acid. There was a significant decrease in wound closure at all tested concentrations of araçá-boi extract and trans-cinnamic acid compared to the control group cells (*p* < 0.0001).

Cutaneous melanoma is an aggressive skin tumor primarily characterized by its high metastatic potential and low survival rate [60]. Metastasis is a multifaceted biological process encompassing invasion, migration, and intravasation into the bloodstream, enabling tumor cells to disseminate and establish colonies in organs distant from the primary tumor site [61]. Recently, several studies have demonstrated that extracts from food matrices rich in phenolic compounds reduce cell migration in melanoma tumor cells [62,63,64]. The results of the present study demonstrated that araçá-boi extract, which is rich in phenolic compounds such as trans-cinnamic acid, induced a significant reduction in wound closure in melanoma cells at all tested concentrations. This finding suggests the potential antimetastatic activity of the araçá-boi extract, as cellular migration is a crucial step in the metastasis process. Additional experiments were performed, to determine the specific contribution of trans-cinnamic acid to the inhibition of cellular migration. When administered alone, trans-cinnamic acid resulted in a significant reduction in cellular migration. These data imply that trans-cinnamic acid may be one of the main contributors to the reduction in melanoma cell migration observed in the araçá-boi extract. The phenolic compounds present in plant extracts can inhibit the migration of melanoma tumor cells by modulating signaling pathways (MAPK/ERK, PI3K/Akt, NF-κB, JAK/STAT, and TGF-β) and enzymes responsible for degrading the extracellular matrix, including matrix metalloproteinases and integrins [65]. Furthermore, dietary polyphenols can act on epigenetic modulation and gene expression related to signaling pathways and key cellular events in cancer [13]. For example, Isacescu et al. [65] presented a comprehensive overview of polyphenol-regulated miRNAs and their modulatory impact on cellular processes involved in melanoma development, including cell growth arrest, apoptosis, epithelial-to-mesenchymal transition, proliferation, invasion, migration, metastasis, and tumor growth. The identification of natural compounds with antimetastatic activity is of great clinical relevance, especially for melanoma, a type of cancer known for its high metastatic rate and resistance to conventional treatments [66]. Thus, araçá-boi extract may represent a promising candidate for the development of new antimetastatic therapies. However, additional studies are needed to fully elucidate the mechanisms of action and to evaluate the efficacy and safety of this extract and its compounds in animal studies and clinical trials.

#### 3.2.4. Detection of Cellular Reactive Oxygen Species (ROS)

**Figure 4:**
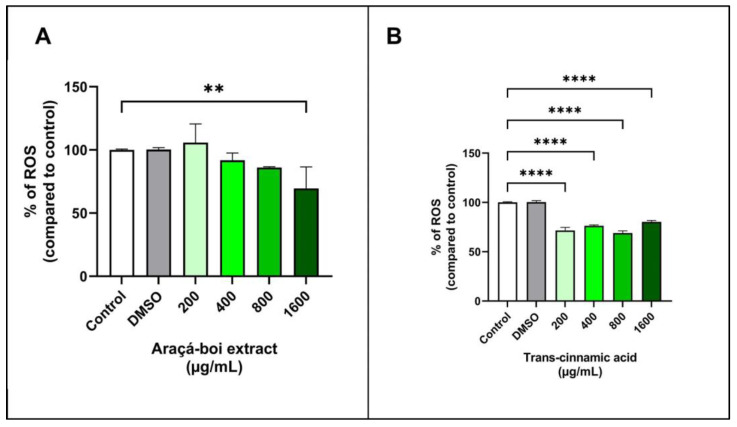
Detection of reactive oxygen species (ROS) levels in tumor melanoma cells treated with araçá-boi extract (**A**) and trans-cinnamic acid (**B**) after 24 h of treatment. CT = control group cells. All experiments were performed independently three times and in three replicates. Data are presented as mean ± standard deviation. Statistical analysis: one-way ANOVA followed by a post hoc Dunnett’s multiple comparisons test. Values with *p* < 0.05 were considered statistically significant. ** (*p* < 0.01) and **** (*p* < 0.0001).

The ROS detection assay is widely used to assess the presence of various reactive oxygen species (ROS) within cells, including hydrogen peroxide (H_2_O_2_), hydroxyl radicals (^•^OH), superoxide anions (^•^O_2_^−^), singlet oxygen (^1^O_2_), peroxyl radicals (ROO^•^), and hypochlorite (OCl¯). The assay uses a non-fluorescent marker (H_2_DCF-DA) that is oxidized by ROS, resulting in the formation of DCF (2′,7′-dichlorofluorescein), which is highly fluorescent [67]. Figure 4 shows the ROS levels in tumoral melanoma cells (SK-MEL-28) treated for 24 h with araçá-boi extract and trans-cinnamic acid. Araçá-boi extract significantly decreased ROS levels only at the highest concentration tested (1600 µg/mL) compared to the control group (*p* < 0.01) (Figure 4A). On the other hand, there was a significant decrease in ROS levels at all tested concentrations of trans-cinnamic acid compared to the control group cells (*p* < 0.0001) (Figure 4B).

ROS, such as hydroxyl radicals, superoxide anions, and singlet oxygen, are inevitable byproducts of cellular metabolism and are essential for various physiological functions such as cell signaling, proliferation, and cell death [68]. Under normal conditions, cells maintain a redox balance in which the generation and elimination of ROS are balanced, thereby preventing oxidative damage. However, tumor cells often exhibit elevated levels of ROS, leading to an imbalance in redox homeostasis and oxidative stress. Oxidative stress can promote DNA mutations and damage biomacromolecules, contributing to uncontrolled cell growth and resistance to apoptosis [13,69,70]. Phenolic compounds, known for their antioxidant and pro-oxidant properties, play a crucial role in modulating stress [71]. At low concentrations, they act as antioxidants, protecting cells against oxidative damage and inhibiting tumor growth. At higher concentrations, they function as pro-oxidants, increasing the production of ROS, overwhelming the antioxidant defenses of tumor cells, and inducing cell death [13,56,71,72].

Our findings demonstrate that araçá-boi extract reduces ROS levels in melanoma tumor cells at the highest concentration. In contrast, trans-cinnamic acid markedly reduced ROS levels at all concentrations tested. These data suggest that high concentrations of araçá-boi extract are necessary to exert antioxidant effects in melanoma tumor cells, possibly due to the increased levels of trans-cinnamic acid present in the extract. Furthermore, other phenolic acids and flavonoids present in the extract may interact synergistically or antagonistically, influencing ROS levels. Phenolic compounds can reduce free radical levels and decrease oxidative stress via several mechanisms. They act directly as antioxidants and neutralize ROS such as hydrogen peroxide (H_2_O_2_) and free radicals such as superoxide anions (^•^O_2_^−^) and hydroxyl radicals (^•^OH) [73]. These phytochemicals can also act as a chelator of transition metals, such as iron and copper, which catalyze free radical formation via the Fenton cycleby binding to these metals, phenolic compounds prevent the formation of hydroxyl radicals and other free radicals [74,75]. Additionally, they can enhance the expression or activity of endogenous antioxidant enzymes such as superoxide dismutase, catalase, and glutathione peroxidase, which help neutralize free radicals and reduce oxidative stress [76,77]. Phenolic compounds can modulate cellular signaling pathways, contributing to protection against oxidative damage, and consequently, DNA damage, apoptosis control, cell-cycle regulation, senescence, and cell fate [78]. The reduction in ROS levels observed with araçá-boi extract and trans-cinnamic acid demonstrates that they could act as antioxidants in tumor cells and protect them against cellular damage and consequently maintain their viability. On the other hand, the reduction in oxidative stress can indirectly affect signaling pathways and potentially inhibit cell migration, as observed above. Thus, it was not possible to clarify the cellular signaling pathway that explains the exact mechanism by which the araçá-boi extract and trans-cinnamic acid exhibit an antitumor effect on melanoma cells. These results underscore the need for additional studies to fully elucidate the role of phenolic compounds present in the extract, the specific mechanisms involved in modulating ROS levels in melanoma cells, and their impact on tumor cell viability and migration.

#### 3.2.5. Expression of Caspase-3 and NLRP3 in Melanoma Cells

**Figure 5:**
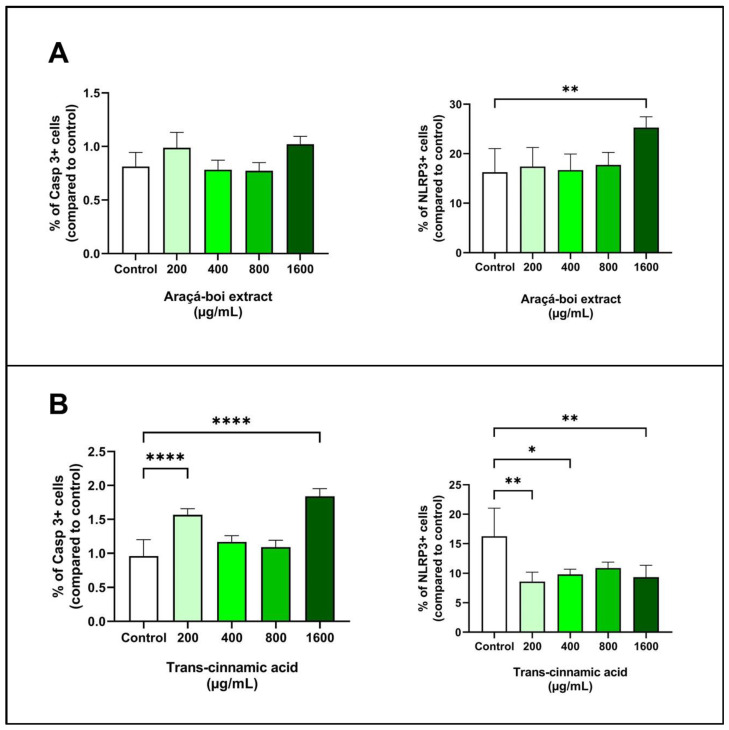
Expression of caspase-3 and NLRP3 in melanoma cells treated with araçá-boi extract (**A**) and trans-cinnamic acid (**B**). CT = control group cells. All experiments were performed independently three times and in three replicates. Data are presented as mean ± standard deviation. Statistical analysis: one-way ANOVA followed by a post hoc Dunnett’s multiple comparisons test. Values with *p* < 0.05 were considered statistically significant. * (*p* < 0.05), ** (*p* < 0.01), and **** (*p* < 0.0001).

Caspases and inflammasomes play critical roles in regulating apoptosis and the inflammatory response in tumor cells, including caspase-3 and NLRP3 inflammasome [79]. Figure 5 presents the effect of araçá-boi extract and trans-cinnamic acid on caspase-3 and NLRP3 expression in melanoma tumor cells after 24 h of treatment. Araçá-boi extract did not show a significant difference in caspase-3 expression compared to the control group cells (*p* > 0.05) (Figure 5A). However, an increase in NLRP3 expression was observed at the highest concentration tested (1600 µg/mL) compared to the control group (*p* < 0.01) (Figure 5A). Trans-cinnamic acid at concentrations of 200 and 1600 µg/mL showed a significant increase in caspase-3 expression (*p* < 0.0001) (Figure 5B). Conversely, trans-cinnamic acid strongly reduces NLRP3 expression at concentrations of 200, 400, and 1600 µg/mL (*p* < 0.05) (Figure 5B).

Caspase-3 is a crucial protease in the execution of apoptosis in tumor cells, and its increased expression is associated with greater activation of apoptotic pathways, which can lead to programmed cell death [79,80]. The activation of caspase-3 is a common endpoint of both the intrinsic and extrinsic apoptotic pathways. The intrinsic pathway is regulated by internal signals from the cell and is often mediated by the release of cytochrome c from the mitochondria and subsequent formation of the apoptosome. The extrinsic pathway is activated by external ligands, such as the FAS and TRAIL ligands, which trigger the activation of executioner caspases-3, leading to cell death [80]. Plant phenolic-rich extracts and isolated phenolic acids, such as rosmarinic acid and curcumin, have been associated with an increase in caspase-3 expression in melanoma tumor cells due to their ability to modulate apoptosis-related cell signaling pathways. These compounds can directly activate apoptotic pathways or influence the expression of pro-apoptotic proteins, leading to the activation of caspase-3 and the subsequent induction of apoptosis [55,56,81]. In our study, we did not observe a significant increase in caspase-3 expression in melanoma tumor cells treated with araça-boi extract, although this modulation occurred with trans-cinnamic acid at certain concentrations. Even though the extract contains trans-cinnamic acid as a major compound, the presence of other phenolic acids and flavonoids may interfere with its ability to induce caspase-3 expression. Moreover, the specific concentrations of these compounds in the extract may not be sufficient to activate the apoptotic pathways responsible for caspase-3 expression. On the other hand, a high concentration of isolated trans-cinnamic acid might favor its interaction with apoptotic pathways, suggesting a potential induction of apoptosis. Thus, the complexity of the extract’s composition and the interactions between different phenolic compounds and flavonoids may explain why the araçá-boi extract did not modulate caspase-3 expression compared to the control, while trans-cinnamic acid showed positive effects.

The NLRP3 inflammasome (NOD-like receptor pyrin domain-containing 3) is a protein complex involved in the regulation of inflammation and immune response, particularly through its role in the formation of inflammasomes. These inflammasomes, when activated, can lead to the maturation and release of pro-inflammatory cytokines like IL-1β and IL-18, contributing to innate immunity and an inflammation-related mode of the programmed cell death process called pyroptosis [82]. Additionally, NLRP3 is indirectly associated with apoptotic pathways. Both apoptotic initiator caspases (such as caspase-9) and executioner caspases (such as caspase-3, 6, and 7) are important for the activation of the NLRP3 inflammasome [83]. Because it can be activated by multiple signals, NLRP3 inflammasomes play an important role in the development of a variety of tumors [84,85]. Several phenolic compounds from different natural sources and medicinal plants have been reported to target NLRP3 and exert beneficial effects against NLRP3 inflammasome-related diseases, including cancer [86,87]. In our study, araçá-boi extract increased NLRP3 expression at the highest concentration tested, whereas trans-cinnamic acid reduced its expression. Despite the extract containing trans-cinnamic acid, several other phenolic acids and flavonoids are present, and thus, the extract’s activity may be influenced by interactions among these compounds. As mentioned above, phenolic compounds may exhibit synergistic and antagonistic effects on biological activities. Therefore, the diverse array of compounds in the araçá-boi extract may explain why it increased NLRP3 expression compared to the control. On the other hand, the reduction of NLRP3 expression by trans-cinnamic acid suggests that this compound may inhibit the activation of the NLRP3 inflammasome, possibly by interfering with specific signaling pathways, including antitumor immunity, cell death, proliferation, angiogenesis, and metastasis [88,89]. Recent evidence suggests that upregulation of the NLRP3 inflammasome may aggravate inflammatory responses in melanoma. Therapies that inhibit the NLRP3 inflammasome can block melanoma migration by suppressing the secretion of IL-1β and IL-18 cytokines and/or activating natural killer cells [84,90]. Thus, trans-cinnamic acid has emerged as a potential therapeutic compound for the treatment of metastatic melanoma. This complexity underscores the importance of further studies to better understand the role of the crude araçá-boi extract and each phenolic compound separately in the extract for more targeted therapeutic applications.

## 4. Conclusions

For the first time, we investigated the effects of an extract obtained from araçá-boi fruit on SK-MEL-28 melanoma cells regarding their viability, cell migration, oxidative stress, and expression of proteins related to apoptosis and inflammation. The HPLC-DAD analysis of araçá-boi extract identified and quantified eleven phenolic compounds, comprising seven phenolic acids and four flavonoids. Phenolic acids represented approximately 56%, while flavonoids accounted for 44% of the total phenolic compounds. Trans-cinnamic acid was the main phenolic compound identified in the araça-boi extract and, therefore, was used alone to verify its contribution to biological activities. Both araçá-boi extract and trans-cinnamic acid treatment significantly reduced cell viability and inhibited cell migration. These results are a promising outcome, indicating potential inhibition of the progression, invasion, and metastasis of melanoma cells by the araçá-boi extract and its main phenolic compound. These findings suggest that the action of the araçá-boi extract may be attributable to the presence of trans-cinnamic acid.

The antioxidant activity of araçá-boi extract and trans-cinnamic acid can be attributed to the reduction in ROS levels. Regarding the evaluation of proteins related to apoptosis and inflammation, the extract did not modulate caspase-3 expression but increased NLRP3 expression. In contrast, trans-cinnamic acid increased caspase-3 expression and reduced NLRP3 expression. This indicates that trans-cinnamic acid can suppress melanoma through pro-apoptotic and anti-inflammatory pathways.

This discovery indicates that araçá-boi extract and trans-cinnamic acid possess antiproliferative, anti-migration, and antioxidant activities and the ability to modulate protein expression related to apoptosis and inflammation in melanoma cells. Although trans-cinnamic acid was identified as a key component of the araçá-boi extract, the results indicate that other phenolic compounds and non-phenolics from the araçá-boi extract may also contribute to the observed effects, particularly in the specific modulation of analyzed proteins.

Thus, the ability of trans-cinnamic acid to reduce oxidative stress and stimulate anti-migratory, pro-apoptotic, and anti-inflammatory effects may be a complementary tool or promising agent for the prevention and management of melanoma. Despite some positive effects of araçá-boi extract (e.g., reduced cell viability and anti-migratory activity) in the management of melanoma, the underlying mechanisms still need to be elucidated. Further studies to understand the specific molecular mechanisms and validate these effects in in vivo models are crucial for advancing research in this area.

## Figures and Tables

**Table 1:** Total phenolic content, condensed tannin content, and antioxidant activity by TEAC, FRAP, and ORAC methods from araçá-boi extract.

Assay	Araçá-Boi Extract
TPC (mg GAE/g extract dw)	12.16 ± 0.38
Condensed tannins (mg CE/g extract dw)	5.76 ± 0.07
TEAC (μmol TE/g extract dw)	102.51 ± 1.16
FRAP (μmol TE/g extract dw)	150.77 ± 4.37
ORAC (μmol TE/g extract dw)	583.81 ± 17.67

dw, dried weight; CE, catechin equivalents; GAE, gallic acid equivalents; ORAC, oxygen radical absorbance capacity; TE, Trolox equivalents; TEAC, Trolox equivalent antioxidant capacity; TPC, total phenolic content.

**Table 2:** Content of individual phenolic compounds obtained by HPLC-DAD method in araçá-boi extract.

Class	Compound	Araçá-Boi Extract (µg/g Extract dw)
Phenolic acids	2,5-Dihydroxybenzoic acid (gentisic acid)	54.94 ± 0.86
	3,4-Dihydroxybenzoic acid (protocatechuic acid)	n.d.
	3,5-Dihydroxybenzoic acid (α-resorcylic acid)	n.d.
	4-Hydroxybenzoic acid	2.56 ± 0.11
	Benzoic acid	n.d.
	Caffeic acid	n.d.
	Chlorogenic acid	n.d.
	Ferulic acid	6.71 ± 0.19
	Gallic acid	73.93 ± 0.18
	*p*-Coumaric acid	28.09 ± 0.49
	Sinapic acid	n.d.
	Syringic acid	113.07 ± 0.74
	Trans-cinnamic acid	155.79 ± 0.32
	Vanillic acid	n.d.
	*Total phenolic acids*	435.09 ± 2.19
Flavonoids	Apigenin	n.d.
	Apigenin-7-*O*-glucoside (apigetrin)	n.d.
	Apigenin-8-*C*-glucoside (vitexin)	n.d.
	Catechin	n.d.
	Epicatechin	n.d.
	Hesperetin	1.15 ± 0.07
	Kaempferol	n.d.
	Kaempferol-3-*O*-glucoside (astragalin)	62.61 ± 0.63
	Luteolin	n.d.
	Myricetin	n.d.
	Naringenin	n.d.
	Procyanidin A2	n.d.
	Procyanidin B1	n.d.
	Procyanidin B2	n.d.
	Quercetin	n.d
	Quercetin-3-*O*-galactoside (hyperoside)	151.32 ± 1.86
	Quercetin-3-*O*-rhamnoside (quercetrin)	116.27 ± 0.27
	Quercetin-3-*O*-rutinoside (rutin)	n.d.
	Vitexin-2″-*O*-rhamnoside	n.d.
	*Total flavonoids*	331.35 ± 2.49
	*Total phenolic compounds*	766.44 ± 4.53

dw, dried weight; n.d.: not detected.

## Data Availability

The authors confirm that data supporting the findings of this study are available within the article, further inquiries can be directed to the corresponding author.
